# Law enforcement responses to mental illness in rural Uganda: A qualitative study of practices, challenges, and opportunities for training

**DOI:** 10.1371/journal.pmen.0000622

**Published:** 2026-06-01

**Authors:** Yang Jae Lee, Kayera Sumaya Nakaziba, Grace Agwang, Eden Sweet, Dana Ragoonanan, Coco Steel, Maggie Han, Travor Nkolo, Ruth Namugaya, Rauben Kazungu, Robert Rosenheck, Alexander C. Tsai

**Affiliations:** 1 Department of Psychiatry and Behavioral Sciences, University of Washington, Seattle, Washington, United States of America; 2 Department of Global Health, University of Washington, Seattle, Washington, United States of America; 3 Empower Through Health, Jinja, Uganda; 4 Scripps College, Claremont, California, United States of America; 5 Frank H. Netter M.D. School of Medicine at Quinnipiac University, North Haven, Connecticut, United States of America; 6 University of California, Berkeley, California, United States of America; 7 School of Public Health, Yale University, New Haven, Connecticut, United States of America; 8 Cavendish University, Kampala, Uganda; 9 Makerere University, Kampala, Uganda; 10 Department of Psychiatry, Yale University, New Haven, Connecticut, United States of America; 11 Center for Global Health and Mongan Institute, Massachusetts General Hospital, Boston, Massachusetts, United States of America; 12 Harvard Medical School, Cambridge, Massachusetts, United States of America; 13 Department of Epidemiology, Harvard T.H. Chan School of Public Health, Boston, Massachusetts, United States of America; PLOS: Public Library of Science, UNITED KINGDOM OF GREAT BRITAIN AND NORTHERN IRELAND

## Abstract

For many people with serious mental disorders, the carceral system often serves as the first point of contact in a mental health crises, yet little is known about how law enforcement personnel in low resource settings, for example, in rural Uganda, recognize and respond to such crises. We conducted 84 in-depth qualitative interviews with individuals representing five groups involved in law enforcement in rural Uganda: Local Council I Chairpersons, Local Council 1 Secretary for Defense, Prisons Officers, Local Defense Personnel, and Criminal Investigation Directorate (CID) officers. Participants were recruited through purposive and snowball sampling across two districts in eastern Uganda. Data were analyzed using the framework method. Participants described a common trajectory. Mental health problems were identified through visible behaviors or alerts from family or neighbors. Officers tried to de-escalate crises before arranging referral, whether informal (i.e., through local leaders) or formal (e.g., health care or criminal justice facilities). They identified numerous barriers, including the need to transport patients long distances, lack of availability of medications, and stigma. Many expressed compassion for people with mental illness, but several described behavioral enactments of stigma, including community vigilantism. They proposed concrete interventions, including brief role-tailored training, structured family involvement, checklists, and supporting basic needs.The Ugandan law enforcement apparatus is commonly called upon to address mental health crises but lack standardized guidance or resources. Psychoeducation and brief role-based training could potentially improve safety and continuity of care.

## Background

Mental illnesses are among the leading causes of disability worldwide, and in low- and middle-income countries (LMICs) the treatment gap often exceeds 90% [1]. Globally, law enforcement officers are often frontline responders to mental health crises and encounter people in acute distress before healthcare professionals [2]. This dual role of maintaining public order and managing complex health emergencies has drawn concern because encounters with law enforcement carry elevated risks of escalation, injury, or incarceration [3,4]. In high-income settings, specialized co-response and community-based Crisis Intervention Teams have been developed to mitigate these risks [5]. However, comparable structures remain relatively rare in low- and middle-income countries (LMICs).

In Uganda, systemic constraints intensify these challenges. There is only one psychiatrist and few other professionals per 1 million people, and with less than 1% of the country’s health budget allocated for mental health services [6,7]. Mental healthcare centers around national and regional referral hospitals, located in urban centers [6]. People in rural districts face long travel distances, delays in assessment, and limited continuity of care [8]. In practice, law enforcement officers, especially outside the capital, Kampala, are often at the front lines of crisis response, and they have limited specialist backup, training, and protocols for referral and follow-up. These gaps are magnified inside carceral settings. Studies in Ugandan prisons report a high prevalence of common and severe mental disorders, which are associated with recidivism [9,10], and broader evidence suggests that many individuals with unmet mental health needs are identified only after arrest or during detention [11]. These findings underscore the importance of interactions with law enforcement personnel for recognition, safety, and linkage to care.

The broader regional picture underscores how system capacity intersects with law enforcement. In Nigeria, a study of 219 police officers found predominantly negative attitudes toward people with mental illness, with high authoritarian and stigmatizing views and general resistance to community-based care initiatives [12]. Community-level accounts in Uganda further describe harsh stigma and even violence, sometimes normalized within everyday life, with many individual attributing mental illnesses to supernatural causes or moral failings [13–18]. While expanded access to universal primary education has not reduced HIV stigma in the Ugandan population as a whole [19], studies in other settings elsewhere in Africa show that higher education and more progressive cultural beliefs are associated with reduced mental illness stigma among police officers, suggesting an opportunity for targeted sociocultural and educational interventions in the region [20,21].

Given this context of law enforcement’s dual mandate, centralized specialist care, plural beliefs, material constraints, and the high mental health prevalence within carceral settings, there is a pressing need to understand how rural Ugandan law enforcement and community security actors define mental illness, make referral decisions, and identify training and system needs both in the community and in places of detention. Understanding their mental health literacy, attitudes, and current practices, along with the barriers that structure those practices, can inform context-specific training and co-response pathways, guide design of simple, scalable referral tools, and identify where health system strengthening would have an impact. The present study addresses these gaps by eliciting the perspectives of law enforcement and community security actors in rural Uganda to inform a feasible coordination of law enforcement and mental health services.

## Methods

### Ethics statement

This research study was approved by the Institutional Review Boards of The AIDS Support Organization, Uganda (TASO-2023–222) and Yale University (2000034605). In accordance with national guidelines, we received approval to conduct the study from the Uganda National Council of Science and Technology (SS1860ES).

### Study site

This study was conducted in the Buyende and Kamuli districts, both located in a rural region of eastern Uganda, with approximately 400,000 and 500,000 people, respectively [22]. Previous studies in the area have shown high mental illness stigma, pluralistic pathways for physical and mental healthcare, and community beliefs attributing mental illness to supernatural causes or moral failings [16,23,24]. Most residents engage in subsistence farming. These districts were selected because they represent the rural eastern Ugandan context where the research team has established community partnerships through Empower Through Health (ETH), a NGO, enabling culturally informed recruitment and data collection.

### Study design and data collection

We conducted 84 in-depth interviews (IDIs) with five groups of law enforcement and community security actors: Local Council I Chairpersons (n = 19), LC1 Secretary for Defense (n = 12), Prisons Officers (n = 20), Local Defense Personnel (n = 15), and Criminal Investigation Directorate (CID) officers (n = 18). Interviews lasted between 20 and 60 minutes to explore mental health literacy, attitudes towards individuals with mental illness, current practices, and willingness to learn about mental health among individuals associated with the local security and justice ecosystem. The five law enforcement categories were selected, in consultation with members of local leadership and community stakeholders, based on their roles in community security, governance, and crime prevention and to ensure representation of the perspectives across different levels of law enforcement present in rural Uganda. Interviews were conducted between July 8^th^ 2024 and August 2^nd^ 2024. We used purposive and snowball sampling to recruit participants aged 18 years or older who belonged to one of the five categories and who were capable of providing written informed consent. Within each category we sought heterogeneity in rank, tenure, and typical duties. Interviews were conducted at private settings of the participants’ choosing, including participants’ homes or offices.

Local Council Chairpersons (LC1) are elected heads of villages, the lowest level of local government in Uganda. They act as intermediaries between community members and higher government authorities. Their key roles include maintaining peace, overseeing local administration, mediating disputes, verifying community members’ identities, and supporting local development initiatives. LC1 Secretary for Defense are responsible for overseeing and coordinating local security efforts and when necessary, mobilizing community responses to local crime at village level. Prisons officers work exclusively within correctional facilities and are tasked with managing inmates, ensuring security within the facilities, and managing rehabilitation programs. Local Defense Personnel are civilian volunteers trained to assist police and local authorities in crime prevention and public order. They assist in community policing, crime prevention, neighborhood patrols, acting as the link between the community and law enforcement. Lastly, Criminal Investigation Directorate (CID) officers are sworn law enforcement officers that are involved in investigating active crimes, apprehending suspects, and enforcing legal statutes.

Our team, which consisted of Ugandan and American investigators, collaboratively developed a semi-structured interview guide consisting of open ended questions designed to elicit study participants’ attitudes towards and experiences with mental health encounters, current practices, perceived training needs, barriers/facilitators across community and carceral settings, and mental health literacy. Guides were piloted and refined for clarity and cultural relevance. The study was described to all potential participants who met the inclusion criteria, so that written informed consent could be obtained prior to conducting in-depth interviews. In the case that participants lacked the ability to read and/or write, a fingerprint was used as a signature alternative on the consent form. In accordance with local etiquette, participants were provided with a transportation reimbursement (20,000 Ugandan Shillings, or approximately $6 in U.S. dollars at the time the study was conducted) at the conclusion of their interviews. Amount of transport reimbursement is consistent with standard rates for transportation reimbursement in research studies conducted in this region and was determined in consultation with the Institutional Review Board of The AIDS Support Organization (TASO) in Uganda.

### Data analysis

All interviews were audio recorded. Audio files were then utilized to develop verbatim written transcripts in English. To ensure translation accuracy, bilingual team members reviewed 50% of transcripts against the original audio recording, and discrepancies were resolved through discussion between the reviewer and the original translator. We used an inductive framework method [25], which provided a systematic way of identifying key themes, which were analyzed and organized into a coherent narrative. Each transcript was coded by at least one Ugandan investigator and one American investigator to generate preliminary codes. This cross-cultural coding approach enhanced interpretive validity by bringing complementary cultural and disciplinary perspectives to the data and reducing the risk of culturally bound interpretation. A supervising investigator independently reviewed the transcripts. Discrepancies were resolved by consensus, with arbitration by the supervisor when needed. Coded data were charted into a framework matrix organized by major themes and sub-themes (rows) against participant/group attributes (columns). The team used the matrix to compare patterns within and across actor groups, map typical vs. deviant cases, and finalize themes grounded in the dataset. Memos documented analytic decisions and theme provenance, and we triangulated findings by systematically comparing convergence and divergence in themes across the five actor groups and across community versus carceral settings. This process helped refine theme definitions and enhance the credibility of interpretations. Interviews continued until saturation was reached within each law enforcement group, i.e., no substantive new codes emerged.

### Positionality

Interviews were conducted by trained research assistants from Uganda and the United States, including undergraduate and masters students, and staff members from Empower Through Health (ETH), an organization providing mental healthcare in Buyende District. Each interview was conducted by a team of two trained research assistants - one Ugandan, fluent in English and Lusoga (the local language), and one American. These assistants were bachelor or master-level students who completed a one-week training in qualitative interviewing methods, research ethics, the study protocol, and administration of screening tools prior to data collection. These assistants were fellows in the Global Health Experiential Fellowship [26]. Reflexivity was integrated into the study design through regular team debriefing sessions during data collection, in which interviewers discussed how their positionality and assumptions may have influenced participant responses, and through supervisory review of early transcripts to identify and correct potential leading or biased questioning. Affiliation with ETH, a health-focused NGO, could have resulted in social desirability bias in the data.

## Results

Inductive analysis yielded three overarching themes: (1) mental health literacy, (2) current practices across community and carceral settings, and (3) challenges and proposed solutions. Across interviews, participants described (Theme 1) how they recognized and explained mental illness and what attitudes they held toward affected individuals; (Theme 2) what they reported doing during crises across community and carceral settings; and (Theme 3) constraints that shaped these practices and solutions participants viewed as feasible. Within each theme, we summarize the most common patterns and then illustrate them with selected quotations.

### Theme 1: Mental Health literacy

Mental health literacy emerged as a foundational theme because participants’ beliefs and knowledge about mental illness directly shaped how they recognized and responded to crises at first contact and influenced the referral decisions described in Theme 2. We identified three sub-themes: knowledge, positive attitudes, and negative attitudes.

#### Sub-Theme 1: Knowledge.

Participants described pluralistic explanatory models of mental illness (biomedical, psychosocial, and spiritual) often coexisting simultaneously within a single account. One local leader shared both biomedical and sociocultural/hereditary mechanisms:

*“Most people say that when someone has a mental illness it is due to failure of the blood vessels to deliver blood properly to the brain. Others say it’s because of the family especially traditions present in the clan” – Local Council I Chairperson, 54 year-old man*

Spiritual causation frequently co-occurred with biomedical or infectious explanations, underscoring layered causal logics rather than either or thinking:

*“One, mental illness due to HIV. Second, there is some virus that causes epilepsy. There is fever, it can also lead to mental illness. It could be traditional and sometimes people bewitch each other” - LC1 Secretary for Defense, 51 year-old man*

Officers also emphasized psychosocial stressors (grief, hardship, and substance use) as triggers or amplifiers:

*“Examples of mental illness, like someone with too much stress, someone who has lost his relatives, witchcraft, they also witch you and you start running mad. Then we also have people who are mentally ill with drugs, they take drugs and they are not okay, they end up becoming a problem in their brain” - Prisons Officer, 32 year*-*old man*

Recognition of mental illness commonly relied on observable behaviors, a practical heuristic they often used when deciding whether to intervene:

*“There are people who just cry continuously nonstop. That’s when you know they have a mental illness. There are others who throw stones at people. There are others who sleep outside and not in the house. Others are at the rubbish pits picking dirty things. There are others who can’t tell who their relatives are. There are others who walk in the middle of the road not knowing they can get knocked by vehicles” - Local Defense Personnel, 38 year-old man*

Among participants, knowledge was layered but inconsistent: respondents mixed biomedical terms with spiritual attributions and psychosocial factors.

#### Sub-Theme 2: Positive attitudes.

Across groups, many participants expressed compassionate and non-punitive views toward people with mental illness, emphasizing dignity and solidarity:

*“I first think of the life of that person, because much as that person is mentally ill, he has to be there and always consider this is the end of their life. Today you are in prison. Tomorrow you will be a very important person. I always give them the references like Nelson Mandela and some prominent politicians in Uganda who have ever been in prison. Then, I will build hope on them. At the end of the day, they become our best friends. That’s why we are living with them” - Prisons Officer, 34 year-old man*

Several participants expressed views that individuals with mental illness should not be held accountable for behavior during periods of symptomatic illness prior to receiving treatment:

*“I think after these people get better, they should not be responsible for what they did when they were still ill” - Local Defense Personnel, 53 year-old man*

Some participants believed that individuals with mental illness should not be treated differently from others:

*“In brief these people are just like any other people and they shouldn’t be isolating them or seeing them as a nuisance” - Local Council I Chairperson, 48 year-old man*

Some LC1 Secretary for Defense highlighted shared community responsibility to facilitate care:

*“It is not a crime to become ill. They just need treatment. What the community should do is help the person get treatment” - LC1 Secretary for Defense, 38 year-old man*

Positive attitudes centered on hope for recovery, sympathy for families, non-punitive treatment during illness, and a community role in helping people access care.

#### Sub-Theme 3: Threat to social order.

Despite some compassionate views, negative attitudes and stigma were pervasive. Participants expressed fear and exclusionary sentiments towards people with mental illness including worries about sudden aggression:

*“When I meet a sick person mentally, of course I develop fear. A person who is not 100% thinking upright will beat you, so you must be afraid. He can jump on you and begin beating you without anything. He can disturb you in all conditions, he can beat children” - Criminal Investigation Directorate (CID) officer, 36 year-old man*

Other accounts emphasized perceptions of disability and low social functioning:

*“People with mental illnesses can’t understand. Even during elections they can’t vote. They can’t do anything good in the area” – Local Council I Chairperson, 55 year-old man*

Additionally, law enforcement also suspected malingering to avoid accountability:

*“People fake mental illness in order to be forgiven on that basis” - Criminal Investigation Directorate (CID) officer, 38 year-old man*

Participants endorsed highly stigmatizing views of people with mental illness, including fear of unpredictability, social exclusion, and assumptions of dangerousness or incapacity. Participants then described how these understandings and attitudes shaped what they did during crises, which we report in Theme 2.

### Theme 2: Current practices

Current practices emerged as the second overarching theme, capturing the operational reality of how officers responded once they recognized a potential mental health crisis. This theme connects to mental health literacy (Theme 1) because beliefs shaped initial responses, and to challenges (Theme 3) because resource constraints limited what practices were feasible. We identified four sub-themes: first contact, referral and legal pathways, family and community responses, and outcomes across settings.

#### Sub-Theme 1: First contact & stabilization.

Across interviews, first responses were pragmatic and scene-driven. Officers described quickly gauging risk from presentation and context, attempting conversation if possible, then referring:

*“Most of the cases we get are first entry point screening. I manage them by showing them the truth, talking to them. But if their condition is severe, we do refer to the mental clinics for mental illness because we have levels of referrals.” - Prisons Officer, 29 year-old man*

Temporary separation was commonly used when officers perceived imminent risk or when immediate clinical input was unavailable. Separation was framed as a short, interim step rather than an endpoint, with the goal of creating enough stability to organize transport:

*“First of all, we isolate them, put them in separate places (from the other prisoners). Then we consult experts like psychiatric doctors to examine such a kind of person. After they examine, we take them for treatment”*

*- Criminal Investigation Directorate (CID) officer, 34 year-old woman*

First contact centered on a quick, pragmatic stabilization routine of conversation and separation while arranging evaluation.

#### Sub-Theme 2: Referral & legal pathways.

When officers judged that symptoms or risk warranted clinical input, they described two main referral streams: health facilities (mainly Butabika National Referral Hospital) and, for detainees already in custody, Jinja Main Prison, where psychiatric clinical officers could assess patients and initiate treatment. Butabika was referenced as a definitive site for confirmation of diagnosis or treatment initiation:

*“There is a big hospital in Kampala for the mentally ill people called Butabika. That’s where we can confirm about their illnesses” - Local Defense Personnel, 53 year-old man*

For incarcerated individuals, prisons officers stated that mental health treatment was available at Jinja Main Prison, the largest prison in the area:

*“So now past here, we have in Jinja, that is Jinja main prison where they have the psychiatric clinical officers and they handle them with medication and care for them” – Prisons Officer, 29 year-old man*

Criminal Investigation Directorate officer highlighted the use of Form 24 specifically as a formal process used in the police system for determining an individual’s mental state:

*“That 24 form is a police form which can take a person who they are suspecting to be mental illness to addressing the doctor so that the doctor can interview him. They confirm whether this person has a mental illness or not” - Criminal Investigation Directorate (CID) officer, 54 year-old man*

Although a formal pathway that exists in principle (Form 24, clinician, then facility), it is unevenly implemented and constrained by resources:

*“We don’t have specialists for those people here, who deal. They’re very far. Like if somebody’s mentally sick we send to Jinja. And even sometimes Jinja refers. That’s the problem. We don’t have health facilities for such people.” – Prisons Officer, 37 year-old man*

Referral and legal steps are present but unevenly implemented.

#### Sub-Theme 3: Family & community responses.

Families played a central role in decisions, logistics, and documentation. In incidents involving an alleged crime, officers described involving relatives early to understand the episode context and to gather or verify hospital paperwork indicating treatment status.

“*When someone with mental illness commits a crime we get the family members involved so that we can discuss with them. We do this in order to find out whether the person with mental illness committed it had an episode or not and if the person with mental illness is cured we ask for forms from the hospital. We go through those forms” - LC1 Secretary for Defense, 38 year-old man*

Community pathways frequently began outside of the formal biomedical and legal system. Several accounts emphasized that families often sought traditional healers or religious leaders first:

*“Sometimes people take their patients to the traditional healers for treatment for the local medicines” - Local Council I Chairperson, 48 year-old man*

Local leaders described stepping in with guidance and counseling aimed at reducing conflict and maintaining calm (both within families and in the broader community) while efforts to arrange care were ongoing:

*“I keep on giving guidance and counseling. I also advise them to keep social. Then I also advise the people to be soft with them and never to be so aggressive or rude” – Local Council I Chairperson, 48 year-old man*

At the same time, officers reported that community justice sometimes superseded legal procedures when serious harm was alleged, creating risk and complicating safe transfer to care:

*“The public will kill. If he does something like for instance, killing, rape, they take the law into their hands” - Criminal Investigation Directorate (CID) officer, 38 year-old man*

#### Sub-Theme 4: Outcomes across settings.

Participants described improvements when treatment and structured routine were sustained, particularly in carceral settings where vocational activities were available. Prison Police highlighted these rehabilitative elements as integral to stabilizing behavior and building hope prior to release:

*“Show them the truth we show them they’re important and they can do something. We teach them work like gardening. We teach them. Some of them we teach them vocational courses like bricklaying. At the time they served their sentences and go out, you find that they are becoming different people. They are learning something good” - Prisons Officer, 29 year-old man*

However, participants also acknowledged that outcomes were inconsistent and relapse into mental health crises after release was common:

*“Unfortunately there was a certain prisoner here with a mental problem who was later on released. We thought he was ok. But some like a month ago we again saw him on the road. He was again totally in a very bad state so it is a challenge to tell whether the person is ok or what it is a challenge” - Prisons Officer, 29 year-old man*

These contrasting accounts (short-term stability with program structure versus post-release deterioration) point to the unpredictability of outcomes on release. Participants then described barriers that constrained these practices and proposed solutions they viewed as feasible, which we present in Theme 3.

### Theme 3: Challenges and proposed solutions

Challenges and proposed solutions constituted the third overarching theme, capturing the structural barriers that constrained the practices described in Theme 2 and the concrete, implementable changes participants proposed to address them. We identified three sub-themes: resource constraints, education needs, and proposed solutions.

#### Sub-Theme 1: Resource constraints.

Participants consistently described material limits that shaped what responses were possible. Access to care was often hindered by distance and support. Participants emphasized that getting to a facility was costly and time-consuming, delaying assessment and treatment:

*“The action of going to hospitals has always helped my people but the problem we have is that the hospitals are always so far from my people which is so overwhelming and makes it hard for them to receive treatment. The transportation is not easy” – Local Council I Chairperson, 65 year-old man*

Even when transfers happened, medication access was uncertain with both availability and affordability as routine obstacles:

*“The other challenges are medication. Those people are going now into the other stage of now instructing them to take medicines, getting psychiatric medicines. It’s very expensive and difficult to access” – Prisons Officer, 29 year-old man*

Frontline safety was affected by gaps in basic protective supplies, with officers describing contact without gloves or comparable equipment:

*”So sometimes you don’t have gloves, and you’re forced to carry that kind of stuff. That thing (froth from a person seizing) goes into your hand and you, how do I explain it even? …. You have no gloves. These are some of the challenges we have” – Prisons Officer, 40 year-old woman*

Leaders also highlighted basic needs, such as food, clothing, and follow-up, that were difficult to sustain and intertwined with recovery:

*“We also have a problem of feeding, sometimes you’d wish them to have some good diet, but because of financial problem, you cannot do it” – Local Council I Chairperson, 47 year-old man*

Resource constraints cut across the pathway with costly transport and distance, limited medications, and few specialists, constraining safe stabilization, delaying referral, and complicating ongoing care and support.

#### Sub-Theme: 2 Education.

Participants across all roles expressed a strong desire for mental health education to help with handling people in mental crisis. Some requests centered around recognizing signs of mental illness:

*“We have a less knowledge about mental illnesses so it becomes so hard for us to address these issues. We need more knowledge in the ways we can handle these illnesses.” - LC1 Secretary for Defense, 32 year-old woman*

Others requested learning how to communicate effectively in tense situations:

*“Then also, educating us on how to handle such cases. Or maybe, some ways of identifying the person is, maybe mentally ill.” – Prisons Officer, 31 year-old woman*

Communication emerged as a concrete need, both how to speak with someone in crisis and how to bridge language barriers in a country with over 70 languages:

*“One of them is language. You find that here in prison there is Lusoga so you may find that that person suffering from mental illness doesn’t know another language. He only knows Lusoga and I don’t know it.” – Prisons Officer, 34 year-old man*

Across accounts, participants endorsed wanting education centered around recognition of signs, how to communicate, and advocating for public awareness, with a preference for action-oriented training to better conduct their roles.

#### Sub-Theme 3: Proposed solutions.

Participants suggested community-anchored solutions that build on existing roles and routines. Some participants called for village-level sensitization led by trusted figures in the community with a focus on where to seek care, what treatment is available, and how to engage people safely and non-violently:

*“We should organize outreaches and awareness campaigns through the local chairpersons who can gather people together for the healthcare workers to teach them about mental illnesses.” – Local Defense Personnel, 53 year-old man*

Participants also highlighted the necessity of involving family:

*“I want to see is more information being spread to relatives of people with mental illness. We need to tell them about the hospital, not to give up on their patients completely.” – Local Council I Chairperson, 40 year-old man*

Several participants proposed mechanisms to keep track of people needing follow-up:

*“I request that the government works together with us. We can list all the people with mental illnesses and send that to them so that they can have treatment and services the same way they do to kids about polio and other diseases” – Local Council I Chairperson, 54 year-old man*

Participants also emphasized the need to meet basic needs (food, education) alongside treatment:

*“The people with mental illness also need food, treatment, and also conduct regular follow up visits for the patients with mental illness to find out how their life is going, how they sleep, the clothing they wear. That is the support I would like to see. If the person with mental illness is a young child they need to be helped to go through school” – Local Council I Chairperson, 54 year-old man*

Proposed solutions were concrete and implementable: outreach, simple tracking lists, and basic social supports to sustain recovery.

## Discussion

In this qualitative study of law enforcement and community security actors in rural eastern Uganda, participants described plural explanatory models of mental illness (biomedical, psychosocial, and spiritual) often simultaneously, while relying on behavior-based cues to recognized distress at first contact. Early responses emphasized conversational calming, brief separation, and quick judgments about whether to seek clinical input. Formal pathways for assessment and treatment existed in principle (use of police Form 24, referral to health facilities including Butabika National Referral Hospital, and evaluation by psychiatric clinical officers at Jinja Main Prison for detainees) but were applied inconsistently and shaped by distance, transport, and familiarity with procedures. Families and local norms played a decisive role in pathways to care, with many accounts describing traditional or religious healing prior to biomedical services, the involvement of relatives in documentation and decision-making, and occasional episodes of community justice. Outcomes were mixed: officers noted improvement where medication and structured routines were sustained, especially during detention, but also described relapse after release when continuity of care and social supports faltered. Cross-cutting constraints included transport, treatment availability, staffing, and basic needs. Participants consistently expressed interest in concise, practical education and proposed community-anchored solutions aligned with existing roles.

These findings are broadly consistent with Ugandan and regional work describing plural beliefs about mental illness and healer-first pathways [27–30]. What this study adds is an operational view from law enforcement actors themselves: officers voiced multi-causal explanations, yet their on-scene choices remained largely pragmatic and behavior-based, shaped by what was observable and logistically feasible. From a policy and implementation standpoint, the findings indicate a gap between the existence of formal pathways (e.g., Form 24 and referral options) and their consistent use in rural settings, largely due to transport constraints, distance, and limited mental health capacity. As a result, routine crisis response depends heavily on non-specialist first responders and families, with substantial variability in how cases are managed and transferred to care. This underscores the importance of pragmatic, standardized supports (e.g., brief training, referral directories/checklists, and structured hand-offs) that do not rely on specialist availability at the point of crisis. The clearer effect of beliefs on timing appeared at the family and community level, which aligns with accounts of rural care-seeking [27,28]. The attitudinal ambivalence we observed (rehabilitative language and sympathy alongside intense stigma marked by fear of unpredictability, perceived dangerousness, and occasional suspicions of malingering) mirrors reports from law enforcement [12] and the general community [31,32] in African settings. These findings also speak to Uganda’s mental health policy and practice landscape, where specialist care remains centralized and community-level first responders, including law enforcement, often operate without standardized protocols for mental health encounters. Prior studies linking greater exposure or education with more favorable attitudes are consonant with participants’ appetite for brief, practical training [20,21]. Consistent with this, contact-based and community-anchored stigma interventions in Uganda have shown promising results [31,33,34].

With respect to practice models, first-contact routines in our data resemble *ad hoc* stabilization described in other low-resource contexts and differ from formal co-response or Crisis Intervention Team approaches common in high-income settings [35]. That divergence is understandable given well-documented system features in Uganda (e.g., centralization of specialist services, long distances, high transport costs, and few mental-health specialists) that also help explain variable use of police medical examination forms and other procedural tools [8]. Family roles, community justice, and the mediating influence of local leaders also align with regional literature on community policing and mental health [36,37]. Finally, the prison interface emerged here as a practical detection and treatment node, consistent with studies showing high burden of mental disorders in African prisons and frequent recognition only after arrest [9,11]. The relapse narratives following release echo re-entry work emphasizing how fragile medication continuity and follow-up can be without explicit linkage [9].

A major strength is our elicitation of perspectives across multiple role categories spanning community and carceral settings, allowing the pathway from first contact to detention and release to be described from multiple vantage points. An inductive framework approach and de-identified transcripts supported transparent theme development and cross-case synthesis. Several limitations should be considered. Social desirability is possible given interviewers’ affiliation with a health-focused NGO and the hierarchical context of law enforcement, despite efforts to mitigate this through reflexive practice and consensus coding. Translation into English may have reduced nuance. Finally, qualitative data describe patterns and mechanisms rather than estimating prevalence or causal effects.

Participants suggested modest, context appropriate adjustments that are feasible within current roles and resources. Officers repeatedly asked for brief, role-specific training focused on core recognition signs and red flags, communication and de-escalation (including language), when and where to refer, and what to document. Simple coordination aids (a referral checklist, a local contact directory for health facilities and psychiatric points of contact, and a short guide to completing and using Form 24) could reduce variability without requiring new infrastructure. Participants also highlighted the practical importance of basic logistics such as transport and airtime to move people to care and complete hand-offs, along with protective supplies to support safe engagement. Community-facing messages delivered through local leaders and health workers could address recurring misconceptions about causation and cost, highlight available services, and discourage mob justice. There is evidence that brief, role-specific law enforcement trainings and community-anchored anti-stigma efforts are feasible and improve outcomes in similar settings [21,38]. Given the pattern of relapse after release, a brief, structured discharge-linkage procedure at the carceral–community transition, such as a brief discharge note with follow-up locations and caregiver contacts, and a hand-off to a named clinic or local leader, may help sustain gains achieved during detention. Any such procedure would need to address confidentiality, ensuring that detained individuals consent freely to sharing their information with community-based contacts prior to release.

Logical next steps could include co-designing and piloting a brief, role-tailored training for officers, paired with a community sensitization session led by local leaders and health workers, to assess acceptability, fidelity, and near-term changes in recognition, communication, and referral behaviors. A small toolkit that combines a referral checklist, a local contact directory, and a Form 24 quick guide could be tested for effects on appropriate routing and time-to-assessment. Introducing a minimal discharge-linkage step from prison to community and documenting post-release continuity and relapse would illuminate the transition point that many accounts identified as fragile.

## Conclusion

In rural Uganda, law enforcement and community security actors often function as de facto first responders for people experiencing mental illness. Their practices are pragmatic and behavior-based, variably connected to formal legal and clinical pathways, and strongly shaped by family decisions and community norms. Participants’ own suggestions (concise training aligned to roles, simple coordination tools, modest logistical supports, village-level outreach, and basic discharge linkage) fit the realities they described. Interventions grounded in participants’ suggestions can test whether these supports improve safety, timeliness, and continuity of care at the law enforcement–mental health interface.
